# Antibiotic choices among healthcare professionals for enterococcal bacteremia with patterns of resistance and risk factors of mortality, in settings of poor antibiotic stewardship program — a five-year retrospective cohort study

**DOI:** 10.1186/s12879-023-08498-0

**Published:** 2023-08-06

**Authors:** Jamil Muqtadir Bhatti, Syed Ali Raza, Ayesha Farooq Alam, Yameena Noman Khan, Ali Mala, Irshad Batool, FNU Sameeullah

**Affiliations:** 1 Dr. Ziauddin University Hospital, North Nazimabad, Karachi, Pakistan; 2 Isra University Hospital, Hyderabad, Pakistan; 3 Steward Carney Hospital, Dorchester, USA

**Keywords:** Enterococci, Ampicillin, Vancomycin, Linezolid, Vancomycin-resistant enterococci, antibiotic stewardship

## Abstract

**Background:**

Enterococcal bacteremia has become prevalent in the recent decade, especially in hospitalized patients. Moreover, the rise in resistance patterns against antibiotic drugs regarding enterococci infection, such as cephalosporins, ampicillin and vancomycin, is prevailing. The major driving force behind this is the incongruous use of antibiotics with a minor contribution from environmental stressors which calls for vigilant and prudent administration of evidence-based antibiotics.

**Methods:**

A retrospective study was conducted from January 1 2017 until December 31 2021, at the tertiary care center, Dr Ziauddin Hospital in Karachi, Pakistan.

**Results:**

Our research revealed ampicillin resistance in 87 (63.5%), with an estimated 25 (18.8%) mortality. Male gender 19 (76%) and vancomycin resistance 13 (52%) were associated with increased mortality. Furthermore, appropriate antibiotic therapy reduced the risk of death compared with inappropriate and excessive use of antibiotics 10 (40%) vs. 15 (60%) vs. 20 (80%) respectively. Targeted therapy with amoxicillin/clavulanic acid was associated with lower mortality 1 (4%) and higher discharge rates 34 (32.1%). On Kaplan-Meier survival, targeted therapy with amoxicillin/clavulanic acid was associated with shorter hospital stays and prolonged survival. UTI was found as the most common source of enterococcal bacteremia 57 (41.6%), followed by respiratory 21 (15.3%) and intra-abdominal 13 (9.5%). In 26 (19%) patients, no identifiable source of infection was found.

**Conclusion:**

Vancomycin resistance and male gender were found independent risk factors for mortality. The use of inappropriate antibiotics significantly increases mortality in these patients. The appropriate antibiotic therapy reduces the risk of death. Furthermore, overuse of antibiotics didn’t reduce mortality; instead increased the financial burden and chances of developing multi-drug resistant strains of other organisms by increasing hospital stays of patients.

## Introduction

Hospitalized patients are becoming more susceptible to enterococcal infections [1, 2]. Enterococci are a regular part of human flora but can cause infections if the host’s immune system is weakened [3]. Enterococci has become the third or fourth most frequent reason for bloodstream infection (BSI) in the last decade [1, 2, 4]. Clinical enterococcal infections are becoming increasingly resistant to vancomycin, with 14 to 25% of all enterococcal isolates in North American hospitals resistant to the antibiotic [4–6]. Enterococci species have been recognized as formidable pathogens because of the high fatality rate linked with enterococcal BSI [7–12]. All enterococci are intrinsically resistant to cephalosporins, and the frequency of ampicillin and vancomycin resistance is on the rise in many countries worldwide. Which complicates the treatment for enterococcal BSI. Synergistic drug resistance has become more widespread [9, 13].

The infamous Enterococci species, *E. faecalis* and *E. faecium*, are commonly mistaken for one another and treated therein. *E. faecium* BSI, has been linked to BSI in a more critically ill population of patients, has greater rates of antibiotic resistance, and is linked to greater mortality than *E. faecalis* BSI [7, 8, 14, 15]. BSI of enterococcal origin is frequently linked to infections such as those of the intra-abdominal region, endovascular area, and urinary passage [7, 14, 16–22]. In previous non-selected observational cohort studies, senile age, male gender, hepatic illness, renal derangement, diabetes, hematopoietic transplant, cancer, and previous antibiotic treatment have all been linked to the acquisition of BSI [7, 8, 14, 21, 23].

In several extensive cohort studies, inadequate and late antibiotic administration has been linked to increased mortality [24]. Antibiotic treatment in enterococcal bacteremia is a contentious topic; a few trials have indicated no reduction in mortality with proper antibiotic therapy [25, 26]. Prospective investigations, on the other hand, have shown that adequate antibiotic treatment improves outcomes for both high-level gentamicin-resistant (HLGR) Enterococci species and Vancomycin-Resistant Enterococci (VRE) [15, 27].

Previous studies have grouped several risk factors likely to increase the chances of VRE infection into three brackets: antibiotic use, host elements, and certain hospital constituents. Other surveys in hospital settings have linked VRE infection with lengthened hospital stays, immunocompromised states/neutropenia, steroid users, renal function derangements, increased exposure to antibiotics, especially vancomycin and 3rd generation cephalosporins, and indwelling urinary catheterization. However, Intensive Care Unit (ICU) patients who are critically ill and have limited treatment options are at tremendous peril of acquiring a VRE infection [28].

The goal of this research was to identify enterococcal bloodstream infection resistance patterns and take into consideration additional known and suspected risk factors. Furthermore, the goal was also to look into the antibiotic prescription practices of doctors in settings with poor/inefficient antibiotic stewardship and their impact on outcomes of enterococcal bloodstream infections.

## Materials and methods

### Study design and population

All cases of enterococcal BSI in the adult population at Dr Ziauddin Hospital, a tertiary care multidisciplinary hospital accommodating 300 beds, were retrospectively collected. As a result, eligibility was granted to all patients at our hospital who have tested positive for Enterococcal species in a blood culture between January 1, 2017, and December 31, 2021.

### Exclusion criteria

Cases excluded from the study were patients under 16, those with incomplete medical records, and patients with polymicrobial bacteremia including non-enterococci or other site infection within three days of the blood culture with Enterococci spp.

### Recognition and susceptibility testing

Blood cultures were conducted via the BD/BACTEC/9000 system. Isolates were identified using conventional biochemical studies. According to Clinical and Laboratory Standards Institute (CLSI) guidelines, susceptibility testing for E. species was conducted via Mueller Hinton Agar (MHA) medium employing a modified Kirby-Bauer disc diffusion technique [29].

### Operational definitions

**Enterococcal Bloodstream Infection**; is defined as the seclusion of Enterococci strains in one or more samples of blood culture [9, 12, 15, 26, 30].

**Fever;** is defined as an elevation in body temperature greater than 37.5 °C using an axillary thermometer [31].

**Appropriate antibiotic treatment;** is defined as having all of the following features (i) starting antibiotics within 24 h of the positive blood culture; (ii) the spectrum of antibiotics administered covered the Enterococci spp. susceptibility and was an approved treatment for the enterococcal disease; (iii) the dosage was sufficient; (iv) no absolute contraindications or relevant interactions with other drugs; and (v) antibiotics were continued for a minimum of six days.

**Inappropriate antibiotic treatment;** is defined as having all of the following features (i) a delay in starting antibiotics past the day blood culture results were positive; (ii) the spectrum of antibiotics administered did not cover Enterococci spp. Susceptibility was not an accepted treatment modality for enterococcal infection; (iii) the dosage was inadequate; (iv) antibiotics were continued for less than five days.

**Excessive antibiotic treatment;** is defined as (i) starting more than one adequate antibiotic treatment for enterococcal infections; (ii) starting antibiotics that are not an acceptable treatment for enterococcal infection besides antibiotics covering enterococcal infection.

**Empiric antibiotic;** is defined as the use of antibiotics any time before and 24 h after this blood culture was drawn or before preliminary microbiologic data was available.

**Prescribed antibiotic;** is defined as the use of antibiotics that covered enterococcal species according to the reported drug sensitivity pattern.

### Data collection procedure

A skilled team of researchers examined the medical data to extract demographic profiles and information on hospitalization, such as dates, length of stay, ward, comorbidities, concurrent infections, patient diagnoses, and clinical outcomes. Also, the usage of indwelling catheters, the vital status, the recommended antibiotic, the dosage, and the number of treatment days were all noted.

Data on the clinical and microbiological aspects were kept. Moreover, information on clinical traits and the number of positive blood cultures was logged. In addition to the blood culture sample, biochemical data were gathered and analyzed on the same day. The patient charts were reviewed to identify the infection’s likely source. Outcomes were further compared based on empiric and prescribed antibiotics (antibiotics advised after isolation of enterococcal species). Results were further compared for appropriate, inappropriate, and overuse of antibiotics. We also compared outcomes according to the sensitivity patterns of enterococci. The author used the terms E and P for empirical and prescribed antibiotics, respectively. Similarly, frequencies and percentages were noted for the consumption trends of empiric and prescribed antibiotics. The structured proforma was used to enter all the data.

### Statistical analysis

Participants’ baseline characteristics were compared for laboratory parameters, existing comorbidities, and signs and symptoms. Continuous variables were compared using the student t-test (standard) or the Mann–Whitney U-test (non-parametric). We calculated frequencies and percentages for categorical variables and compared them using Fisher’s exact test or Pearson’s Chi-square test. Survival analysis was done using the Kaplan-Meier curve. In addition, the results were calculated using both univariate and multivariate analysis (multivariate logistic regression). Logistic regression included variables from univariate analysis with a p-value less than 0.05. Given that there was no demise, the author did not include P-amox in the logistic regression. The data were analyzed with IBM SPSS Version 26, and a P-value < 0.05 was judged statistically significant.

## Results

**Fig. 1:**
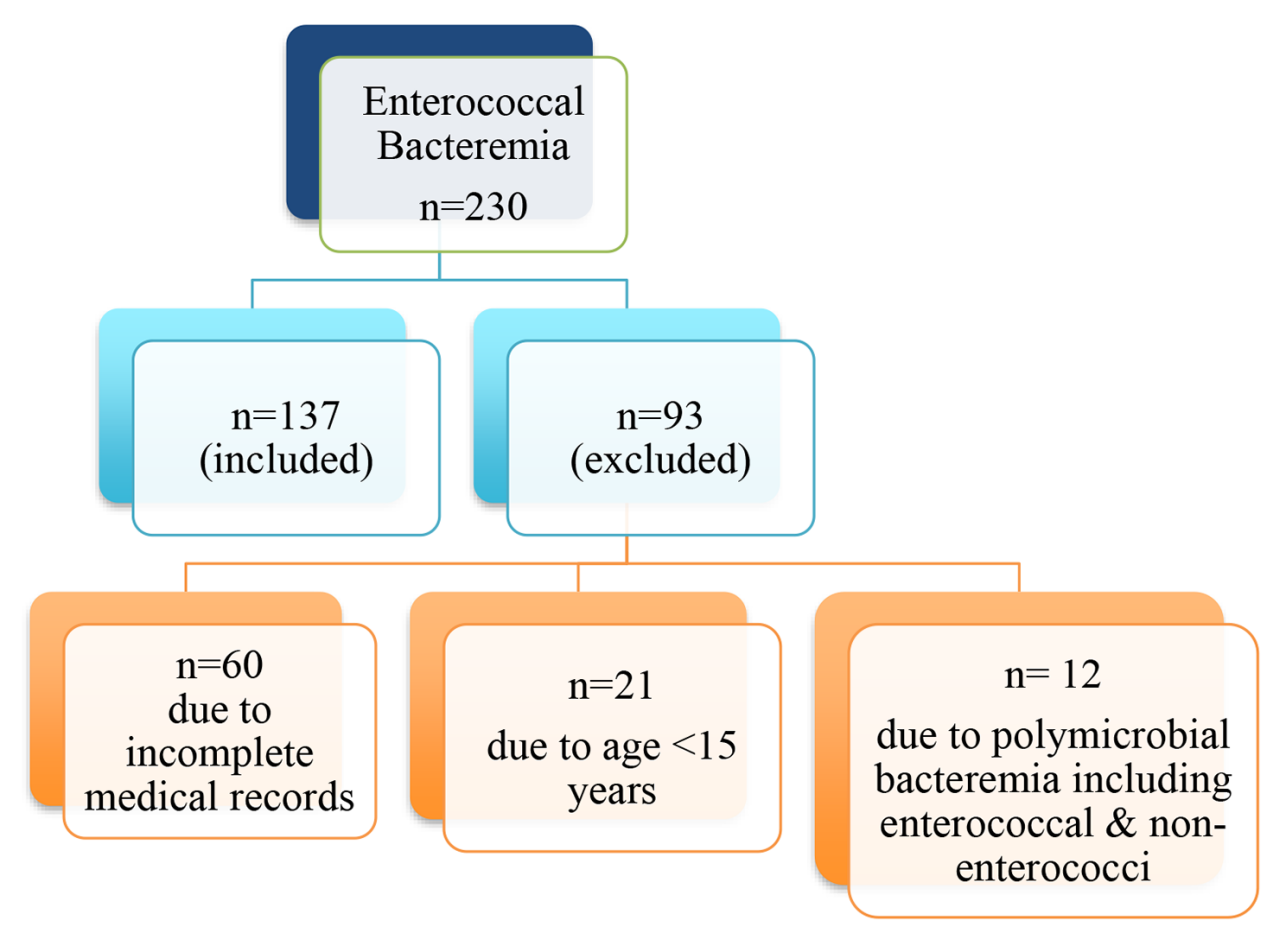
Flow diagram of patients included in the study

A total of 137 patients were included in this study Fig. 1.

Of these, the male/female ratio was roughly the same, with males accounting for 75 (55.5%) and females 62 (44.5%), respectively Table 1. Death was more commonly seen in males with a significant p-value. Although fever was widely observed in surviving patients, there was no statistical significance. The most common comorbid illnesses were hypertension, diabetes, and ischemic heart disease. There was no mortality difference in comorbid conditions. Mortality was significantly higher in patients who needed intensive care unit admission and mechanical ventilation. The most common source of infection was identified as the urinary tract, found in 57 (41.6%). Other common sources included the respiratory tract, found in 21 (15.3%); the intra-abdominal, found in 13 (9.5%); and the pelvic, found in 11 (8%). In 26 (9%) cases, no obvious source of infection was identified. There was no significant mortality difference in the sources of infection. Mortality was lower in enterococci sensitive to beta-lactams and vancomycin, although the difference was insignificant. Similarly, mortality was lower in enterococci resistant to beta-lactams but sensitive to vancomycin with statistically significant p values. However, mortality was highest, with a significant p-value in enterococci resistant to linezolid.

**Table 1:** Demographics of patients with Enterococcal bacteremia

	Total Number = 137 (Percentage)	Non-survivor Number = 25 (Percentage)	Survivor Number = 112 (Percentage)	P value
Mean age in years ± SD	55.3 ± 18.4	58.1 ± 13.9	55 ± 19.3	0.402
Gender				
Male	75 (54.7)	19 (76)	56 (50)	0.015
Female	62 (45.3)	6 (24)	56 (50)	
Diabetes	58 (42.3)	11 (44)	47 (42)	0.512
Hypertension	83 (60.6)	14 (56)	69 (61.6)	0.382
Asthma/COPD	4 (2.9)	0	4 (3.6)	0.442
Solid Organ Malignancy	11 (8.0)	2 (8)	9 (8)	0.678
Hematologic Malignancy	3 (2.2)	1 (4)	2 (1.8)	0.456
Chronic Liver Disease	10 (7.3)	2 (8)	8 (7.1)	0.577
Ischemic Heart Disease	52 (38.0)	9 (36)	43 (38.4)	0.507
Smoking	15 (10.9)	1 (4)	14 (12.5)	0.195
Fever	106 (77.4)	17 (68)	89 (79.5)	0.164
ICU admission	36 (26.3)	15 (60)	21 (18.8)	< 0.001
IMV	18 (13.1)	9 (36)	9 (8)	0.001
Source of Infection				
UTI	57 (41.6)	7 (28)	50 (44.6)	0.095
Intra-abdominal	13 (9.5)	2 (8)	11 (9.8)	0.565
Cutaneous Wounds	9 (6.6)	0	9 (8)	0.153
Respiratory	21 (15.3)	4 (16)	17 (15.2)	0.563
Pelvic	11 (8)	4 (16)	7 (6.2)	0.116
Unknown	26 (19)	8 (32)	18 (16.1)	0.065
Antibiotics Sensitivity Pattern				
Ampicillin + vancomycin (both sensitive)	27 (19.7)	2 (8)	25 (22.3)	0.082
Ampicillin (resistant) + Vancomycin (sensitive)	87 (63.5)	10 (40)	77 (68.8)	0.007
Vancomycin (resistant)	23 (16.8)	13 (52)	10 (8.9)	< 0.001
Antibiotics Usage				
Appropriate	101 (73.7)	10 (40)	91 (81.3)	< 0.001
In-appropriate	36 (26.3)	15 (60)	21 (18.8)	< 0.001
Overused	88 (64.2)	20 (80)	68 (60.7)	0.053

SD—standard deviation; ICU—intensive care unit; IMV—invasive mechanical ventilation; COPD—chronic obstructive pulmonary disease; UTI— Urinary tract infection

Using appropriate antibiotics was associated with significantly lower mortality, while inappropriate antibiotic use was associated with significantly higher mortality. Overused antibiotics were again associated with higher mortality, but there was no statistical significance.

The median (IQR) of laboratory values was correlated with the termination of the patient Table 2. The hemoglobin values in the non-survivors were greater than those in survivors, with a significant p-value of 0.003. Similarly, total bilirubin and creatinine were higher in non-survivors than in survivors, with a significant p-value of.017 and a p-value of.030, respectively.

**Table 2:** Laboratory parameters with survival outcome in Enterococcal bacteremia patients

	Non-survivor Median (IQR)	Survivor Median (IQR)	P value
Hemoglobin (g/dL)	11.6 (10.3–13.4)	10.9 (9.3–12.2)	0.003
White Blood Cells x 10^9^ (/L)	15.6 (7.9–24.2)	12.5 (8.7–17.0)	0.649
Neutrophils (/%)	87.0 (75.0–94.0)	80 (68.0–87.0)	0.979
Lymphocytes (/%)	10.0 (3.0–30.0)	13.0 (7.0–24.0)	0.473
Platelets x 10^9^(/L)	138.0 (91.0–293.0)	198.0 (115.75–281.25)	0.175
Partial Thromboplastin Time (seconds)	31.1 (26.5–34.45)	30.0 (26.8–35.0)	0.384
Prothrombin Time (seconds)	13.8 (11.65–15.0)	12.5 (11.5–14.8)	0.271
International Normalized Ratio	1.29 (1.075–1.470)	1.100 (1.040–1.310)	0.534
Total Bilirubin (mg/dL)	0.820 (0.480 − 1.430)	0.700 (0.313–1.475)	0.017
Serum Glutamic pyruvic transaminase (/L)	27.0 (17.0–61.0)	29.0 (15.0–61.0)	0.055
Gamma-Glutamyl Transferase (IU/L)	92.0 (46.0–203.0)	69.0 (32.0–140.0)	0.491
Alkaline Phosphatase (U/L)	131.0 (90.0–152.0)	117.0 (74.0–194.0)	0.849
Urea (mg/dL)	56.0 (32.0–153.0)	60.50 (28.5–104.5)	0.841
Creatinine (mg/dL)	1.50 (1.00–2.68)	1.4 (0.80–3.40)	0.030
Sodium (mEq/L)	136.5 (130.75–139.25)	136.0 (132.0–139.0)	0.671
Potassium (mEq/L)	4.05 (3.68–5.15)	4.2 (3.7–4.6)	0.197
Bicarbonate (mEq/L)	19.0 (16.0–26.0)	21.1 (18.0–25.0)	0.854
Chloride (mEq/L)	101.0 (98.0–103.0)	103.0 (98.0–106.0)	0.905

IQR- Interquartile range

Different trends of the empiric regimen were used empirically, and these regimens were compared with non-survival outcome Table 3. The most common empiric therapy included ceftriaxone, a combination of a carbapenem and vancomycin, amoxicillin/clavulanic acid, and a combination of carbapenem and linezolid. Patients who received E-amoxicillin/clavulanic acid had significantly lower mortality, while those who received a combination of an E-carbapenem with E-vancomycin had the highest mortality.

**Table 3:** Empiric regimen with non-survival outcome in Enterococcal bacteremia patients

Empirical Antibiotics	Total Number = 131 (Percentage)	Non-survivor Number = 24 (Percentage)	Survivor Number = 107 (Percentage)	P value
Ceftriaxone	30 (21.9)	3 (12.0)	27 (24.1)	0.144
Amoxicillin/Clavulanic Acid	20 (14.6)	0 (0.0)	20 (17.9)	0.013
Carbapenem	12 (8.8)	4 (16.0)	8 (7.1)	0.152
Carbapenem + Vancomycin	23 (16.8)	8 (32.0)	15 (13.4)	0.030
Ceftriaxone + Vancomycin	5 (3.6)	0 (0.0)	5 (4.5)	0.359
Carbapenem + Linezolid	15 (10.9)	2 (8.0)	13 (11.6)	0.458
Linezolid + Colistin	2 (1.5)	1 (4.0)	1 (0.9)	
Carbapenem + Vancomycin + Colistin	3 (2.2)	1 (4.0)	2 (1.8)	0.456
Piperacillin/Tazobactam	5 (3.6)	2 (8.0)	3 (2.7)	0.225
Other Combinations	16 (11.7)	3 (12.0)	13 (11.6)	0.592

As for prescribed antibiotics are concerned, amoxicillin/clavulanic acid was the most commonly used antibiotic 36 (26.3%), followed by combinations of Carbapenem + Vancomycin or Linezolid 17 (12.4%) each, and linezolid prescribed in 16 (11.7%) Table 4. When comparing mortality after cultures reported sensitivity patterns, mortality was highest in patients receiving P-vancomycin with a significant p-value, followed by a combination of P-carbapenems with P-vancomycin and P-colistin. Compared to these, mortality was lowest in P-amoxicillin/clavulanic acid.

**Table 4:** Prescribed antibiotics with survival outcome in Enterococcal bacteremia patients

Antibiotics Prescribed	Total Number = 121 (Percentage)	Non-survivor Number = 19 (Percentage)	Survivor Number = 102 (Percentage)	P value
Amoxicillin/Clavulanic Acid	36 (26.3)	1 (4.0)	35 (31.2)	0.02
Ampicillin	3 (2.2)	1 (4.0)	2 (1.8)	0.456
Carbapenem	10 (7.3)	2 (8.0)	8 (7.1)	0.577
Vancomycin	7 (5.1)	2 (8.0)	5 (4.5)	0.021
Linezolid	16 (11.7)	1 (4.0)	15 (13.4)	0.217
Carbapenem + Vancomycin	17 (12.4)	6 (24.0)	11 (9.8)	0.060
Ceftriaxone + Vancomycin	6 (4.4)	0 (0.0)	6 (5.4)	0.291
Carbapenem + Linezolid	17 (12.4)	2 (8.0)	15 (13.4)	0.362
Carbapenem + Vancomycin + Colistin	5 (3.6)	3 (12.0)	2 (1.8)	0.042
Meropenem + Linezolid + Colistin	4 (2.9)	1 (4.0)	3 (2.7)	0.558

In the multivariate analysis Table 5, only gender and vancomycin resistance were significantly associated with mortality.

**Table 5:** Multivariate analysis in Enterococcal bacteremia patients

	Odds Ratio	Confidence Interval	P value
Age	1.012	0.942–1.088	0.742
ICU Admission	0.185	0.023–1.496	0.114
Intubation	0.507	0.046–5.562	0.578
Male Gender	29.764	1.872–473.128	0.016
Ampicillin & Vancomycin-Resistant Enterococci	0.046	0.003–0.736	0.029
Ampicillin resistant & Vancomycin Sensitive Enterococci	2.232	0.266–18.701	0.459
Hemoglobin (g/dL)	1.253	0.801–1.961	0.323
Creatinine (mg/dL)	1.204	0.736–1.968	0.459
Total Bilirubin (mg/dL)	0.893	0.586–1.361	0.600

ICU- Intensive care unit

**Fig. 2:**
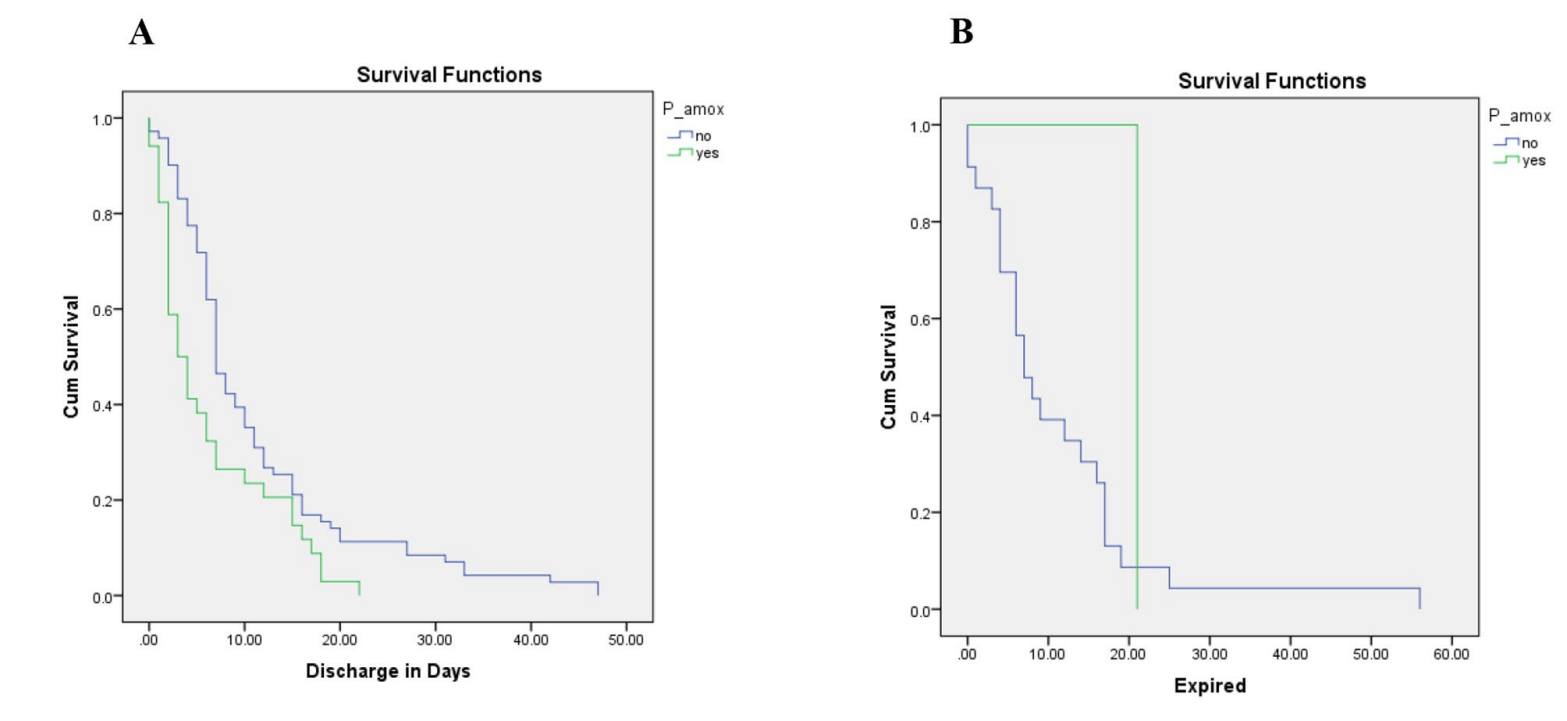
Kaplan-Meier survival of P-amoxicillin/clavulanic acid in Enterococcal Bacteremia patients; (**A**) Time from admission to discharge; (**B**) Time from admission to death P—amox Prescribed amoxicillin/clavulanic acid

According to the Kaplan-Meier survival analysis, patients who received P-amoxicillin/clavulanic acid were discharged earlier (6.32 vs. 11.13 days; Log Rank P = 0.005) (Fig. 2). Similarly, comparing time from admission to death, patients who received P-amoxicillin/clavulanic acid had higher survival, although there was no significant statistical difference (21 vs. 11.22 days; Log Rank P = 0.336). There was no statistical significance here as only one patient receiving P-amoxicillin/clavulanic acid did not survive.

Contrary to that, the survival of patients who received P-Vancomycin remained similar regarding admission to death (11.20 vs. 11.93 days; Log Rank P = 0.886). Similarly, no significant difference was seen in hospital stay, from admission to discharge (11.17 vs. 9.12 days; Log Rank P 0.335).

On the other hand, patients who received P-linezolid survived slightly longer but with no statistical significance when compared from admission to death (13.0 vs. 11.26 days; Log Rank P 0.734). While from admission to discharge, patients who received P-linezolid were discharged home late (12.60 vs. 8.36 days; Log Rank P 0.050). Using survival analysis on appropriate vs. inappropriate antibiotics; it was found that there was no significant difference in overall survival (11.10 vs. 12.0 days; Log Rank P = 0.923). While overuse of antibiotics significantly increases hospital stay from admission to discharge (11.95 vs. 6.14 days; Log Rank P < 0.001).

According to the unadjusted Cox regression model, patients in the P-vancomycin group had a slightly higher risk of death, although the difference was not statistically significant (HR 1.060, 95% CI 0.463–2.424; P 0.891). Similarly, no difference was seen for ICU admission or mechanical ventilation (HR 1.140, 95% CI: 0.541–2.405; P 0.730, HR 1.387, 95% CI: 0.443–4.346; P 0.574, respectively). Using Cox regression on P-amoxicillin/clavulanic acid, there was no statistical difference from admission to death and mechanical ventilation (HR 0.420, 95% CI: 0.055–3.221; P 0.404, HR 2.371, 95% CI: 0.284–19.776; P 0.425, respectively). Using the Cox regression model, the hazard of death was higher in the P-linezolid group (HR 1.183; 95% CI 0.428–3.269; P 0.747). Similarly, ICU admission and mechanical ventilation hazards were higher in patients receiving P-linezolid (HR 1.180; 95% CI 0.481-2,899; P 0.718; HR 1.405; 95% CI 0.50-3.944; P 0.519, respectively).

## Discussion

The study’s key finding was that vancomycin resistance and male gender were independent risk factors for mortality. While the use of inappropriate antibiotics increases mortality, the appropriate use of antibiotics significantly reduces mortality. However, excessive antibiotic use lengthens hospital stays without significantly affecting mortality.

Enterococci have appeared as sources of significant nosocomial and community-acquired illnesses in the past ten years. They were listed as the United States’ second-most prevalent source of hospital-acquired illnesses. Furthermore, enterococci are reported to be the third most pervasive organism in healthcare-associated bloodstream infection in the United States [5].

Multiple kinds of research in Asia have documented *Enterococci* spp. as the fourth most frequent microorganism causing BSI [32]. A systemic analysis also labelled it the third most common gram-positive bacteria in driving community-acquired BSI in Asia and Southeast Asia [33]. Unfortunately, BSI of bacterial and fungal etiology involves more than 200,000 people annually in the United States and is a vital source of morbidity and mortality globally [4].

Over the years, Enterococci have developed resistance to several antibiotics owing to three significant factors: [1] excessive administration of broad-spectrum antibiotics such as penicillin and cephalosporins, which results in the growth of gram-negative intestinal bacterial flora; [2] specific strains adapting and circulating the facets of antibiotic resistance; and [3] innate resistance to some frequently prescribed antibiotics [34]. But its ability to cause disease is strongly linked to the development of VRE and other features of multi-drug resistance (MDR) [35].

This study reported UTI as the most common source of enterococcal BSI, which is similar to findings by McBride et al. [14]. from New Zealand, where most cases of enterococcal bacteremia (52/205, 25.3%) originated in the genitourinary system, a frequency lower than reported in this study. The second most prevalent diagnosis for the cause of bacteremia was an unidentified source (21.5%) [14]. However, the unknown source remains almost identical between the findings of McBride et al. and this review. Similarly, in a study by Caballero-Granado, the most common source of enterococcal BSI was reported as intra-abdominal, followed by intravascular catheters and the urinary tract [10]. Although in their study, 39% of patients had no identifiable source of bacteremia [10]. Our findings are consistent with Ceci et al. [36], who reported the urinary tract as the most common source of enterococcal bacteremia found in 36.4% of patients, followed by vascular catheters and cutaneous infections. However, almost half of the patients (48.5%) in their study had no obvious source of infection. Lark et al. [37] noticed bacteremia that is with no documented origin to a less frequent extent (7.0%); the catheters were associated with 47.0% of the cases, whereas infections of the urinary tract had a smaller frequency (11.0%) after intra-abdominal sites, respiratory infections, and the skin or soft-tissue infections. Nevertheless, a prior investigation in Argentina found a much higher frequency (42.0%) of bacteremia of unknown cause, subsequently followed by respiratory, urinary, cutaneous, and abdominal origin [38].

Conventionally, antibiotics, mainly cell wall inhibitors coupled with aminoglycosides, treat enterococci-related infections [39]. However, the inborn robust nature of Enterococci manifests an atypical capability to confer resistance to multiple categories of medication, including macrolides, β-lactams, tetracyclines, aminoglycosides and fluoroquinolones [39]. Hence, one of the most burdensome tasks for today’s physicians regarding Enterococcal infections is the treatment since these organisms either possess innate resistance or are collectively less susceptible to most antibacterial drugs [40].

Collectively, the current review is established to account for the drift of antibiotic resistance in enterococci over 5 years and the association of inappropriate antibiotic regimens with mortality in enterococcal bacteremia in a tertiary care hospital in Pakistan. Our analysis of enterococcal isolates showed profound ampicillin resistance (78.8%), and less than a quarter were resistant to vancomycin (16.8%), out of which 15.3% had both ampicillin and vancomycin resistance. In comparison, a study conducted in Rawalpindi, Pakistan, in 2012 reported the frequency of VRE to be 11.57% [41]. Furthermore, another study done in Karachi, Pakistan, found that the frequency of VRE was relatively low at just 0.9% [42]. Considering the studies mentioned earlier, a general upward trend is noted in the prevalence of VRE in Pakistan. Our findings support the growing trend of antibiotic resistance among enterococci, as reported in the literature. For example, research on the regional resistance pattern of enterococci found that resistance to vancomycin and ampicillin grew from 14% to 21% in 1997 to 17% and 24% in 1999 (increases of 1% each year) [5]. Another source of worry is the advent of glycopeptide resistance in Latin America, initially found in 1998 at a relatively low incidence (1%) but nearly doubled to 2% in the subsequent year [5]. McBride et al. reported resistance to amoxicillin in 69.0% (20/29) of E. faecium isolates [14]. However, there were no vancomycin-resistant enterococci [14]. Additionally, it was observed that studies from many countries, as well as Pakistan, produced conflicting findings for instance, a study in Eastern India showed all Enterococci isolates to be vancomycin and linezolid sensitive [43]. Moreover, a review conducted in Iran showed that resistance to erythromycin, ampicillin, ciprofloxacin, gentamicin, and vancomycin fluctuated between 2001 and 2016, with vancomycin showing a negligible increase in resistance, while erythromycin and ampicillin showed decreasing trends in resistance [44].

One of the key findings of this study was that approximately one-fourth of the patients (26.3%) had received inappropriate antibiotics. These findings are comparatively lower than the study by Napolitano et al. [45], who reported higher use of inappropriate antibiotics (34.2%). Due to the various study criteria, medical settings, and participant characteristics, it is impossible to compare this inappropriateness rate with earlier studies carried out in Pakistan and other nations. However, despite these variations, greater rates were discovered in two earlier studies, where 33% of antibiotics in a Swiss tertiary care hospital [46] and 32.7% of prescriptions in Australian emergency rooms were deemed inappropriate [47]. Similarly, slightly higher rates of inappropriate prescriptions have been noted in a Dutch university hospital (29.3%) [48].

In contrast, a previous study in the same region discovered much higher rates in medical, surgical, and intensive care units, ranging from 53.8 to 79.8% [49]. These findings imply that there is space for improving adherence to antibiotic prescribing guidelines by putting efficient initiatives into practice. Antibiotic stewardship programs (ASP) are well known to have a favorable effect on antibiotic use. They may enhance hospitalization outcomes, such as a decrease in infectious diseases brought on by multidrug-resistant microorganisms, lengths of stay, readmission rates, and patients’ disability and mortality [50–53]. Another major finding of this study was the overuse of antibiotics. Despite positive cultures with enterococci, after excluding other co-infections, 64.2% of patients were prescribed extra antibiotics with either dual coverage for enterococci or mainly prescribing antibiotics for polymicrobial bacteremia including non-enterococcal infections. The results of a Pakistani multicenter survey on antibiotic stewardship revealed that patients also influence physicians’ prescriptions by compelling them to include an antibiotic to achieve the quickest cure [54]. This finding was similar to studies from Sri Lanka and the United Kingdom [55, 56] that demonstrated how patients influenced physicians to prescribe antibiotics even when they were unnecessary. Without a doubt, the results of the culture and sensitivity tests should be used to guide antibiotic selection. Unfortunately, doctors are forced to overprescribe broad-spectrum antibiotics due to delays in the release of microbiology reports, a lack of confidence on the part of doctors in laboratory results, the limited availability of antibiotics in hospitals, the influence of patients and their families on doctors’ prescriptions, the absence of an efficient program for the stewardship of antibiotics, the fear of losing patients, and the poor financial situation of patients.

Our study gathered isolates, treated empirically with 17 different representative drugs, followed by targeted therapy on the arrival of the sensitivity report. It was noticed that patients who received appropriate empirical treatment were associated with better survival outcomes, regardless of other risk factors for mortality. Mortality in this review was reported at 18.2%, a finding almost identical to McBride et al.‘s conclusions, who noted that the crude 7- and 30-day death rates in their patient group were 13 and 25%, respectively [14]. Others have found crude death rates ranging from 13 to 68% for enterococcal bacteremia [10, 25]. It has been postulated that a significant portion of this mortality is related to causes other than bacteremia, with attributable death rates ranging from 2 to 43% [10]. Suppli et al. observed high enterococcal bacteremia death rates of 26% [57]; Danish research revealed low bacteremia mortality rates of 18% at 30 days and 25% at 90 days [58]. In 398 cases of enterococcal bacteremia, Vergis et al. [15] discovered a 14-day death rate of 19%. In another study, mortality was found to be 37% in enterococcal bacteremia, where the severity of illness and age were independently associated with mortality [26].

Similarly, 39% mortality has been found in another study [59]. According to our findings, the most important variables related to death included gender, the necessity for ICU admission and mechanical ventilation, improper antibiotic administration, using carbapenem and vancomycin alone or in combination, and the isolation of VRE. There is substantial debate about whether vancomycin resistance is a reliable indicator of death in enterococcal bacteremia. According to two studies, VRE infection is not independently related to mortality when illness severity is considered during multivariate analysis [60, 61]. Vancomycin resistance, on the other hand, was discovered to be a standalone predictor of death by another group, which used the presence of shock as a sign of disease severity [12]. In addition, we found that patients with VRE had a much greater fatality rate than those with Vancomycin-Sensitive Enterococci (VSE) in our study, which included a small cohort of patients with VRE bacteremia.

The fact that mortality was significantly decreased when P-amox was given to patients was a key finding of this study. This could be explained by the fact that only a small portion of the patients in this group were critically ill. P-amox was used in just one patient who required invasive ventilatory support and a small number of patients who required ICU admission. P-vanco, on the other hand, was utilised more frequently in patients who required invasive mechanical ventilation and ICU admission. Therefore, to determine whether the use of amoxicillin/clavulanic acid truly contributes to the survival rate, a randomized comparative trial with matched patient backgrounds is necessary, and this study alone does not provide a clear answer.

There are several limitations to this study. First, we didn’t classify the enterococci as health-care-associated or community-acquired. As undoubtedly, infections acquired in healthcare settings are associated with poorer outcomes. Second is the nature of the study and single-centre experience with smaller sample size. Third, due to limited resources, enterococci were not differentiated into *faecalis* or *faecium* species, which may have resulted in study bias. Fourth, due to the lack of a control group, we cannot investigate the attributable mortality or correlate the results to those of bacteremia caused by other organisms. The main strengths of this study included being the first in the region to directly report the antibiotic choices made by physicians for treating enterococcal BSI.

In reality, the best way to counteract antibiotic resistance is by properly administering antibiotics and recognizing these diseases to prevent their occurrence rather than inventing new drugs. Therefore, the first and foremost action is to caution doctors against haphazardly using antibiotics like vancomycin [39].

## Conclusion

The clinical importance and burden of enterococcal bloodstream infections should be considered. Vancomycin resistance and gender were found to be independent risk factors for mortality. The use of inappropriate antibiotics significantly increases mortality in these patients. The use of amoxicillin/clavulanic acid is associated with a reduction in mortality, increased hospital discharge rates, and reduced hospital stay. Therefore, to determine whether the use of amoxicillin/clavulanic acid truly contributes to the survival rate, a randomized comparative trial with matched patient backgrounds is necessary, and this study alone does not provide a clear answer. Furthermore, overuse of antibiotics didn’t reduce mortality; instead, it increased the financial burden and chances of developing multi-drug-resistant strains of other organisms by increasing patients’ hospital stays.

## Data Availability

The datasets used and/or analyzed during the current study are available from the corresponding author on reasonable request.
